# Observational Study on Antibody Response to COVID-19 Vaccines in PAtients with Gastro-Entero-PanCreatic Cancers and NeuroendocrIne NeoplAsms on Systemic TreatmEnts (VACCINATE)

**DOI:** 10.3390/biomedicines11020336

**Published:** 2023-01-25

**Authors:** Alice Laffi, Lorenzo Gervaso, Oriana D’Ecclesiis, Sara Gandini, Agostino Riva, Rita Passerini, Francesca Spada, Stefania Pellicori, Manila Rubino, Chiara Alessandra Cella, Paola Simona Ravenda, Maria Giulia Zampino, Nicola Fazio

**Affiliations:** 1 Division of Gastrointestinal and Neuroendocrine Tumors, European Institute of Oncology (IEO), IRCCS, Via Ripamonti 435, 20141 Milan, Italy; 2 Department of Experimental Oncology, European Institute of Oncology (IEO), IRCCS, Via Ripamonti 435, 20141 Milan, Italy; 3 III Division of Infectious Diseases, ASST Fatebenefratelli Sacco, Luigi Sacco Hospital, 20157 Milan, Italy; 4 Division of Laboratory Medicine, European Institute of Oncology (IEO), IRCCS, Via Ripamonti 435, 20141 Milan, Italy; 5 Oncologic Department, ASST Lodi Hospital, Piazza Ospitale 10, 26900 Lodi, Italy

**Keywords:** COVID, SARS-CoV-2, gastroenteropancreatic cancer, vaccines, neuroendocrine tumor

## Abstract

The coronavirus disease-19 (COVID-19) pandemic dramatically impacted oncological patients’ care. Since the introduction of vaccines and the demonstration of their benefit on frail patients, COVID-19 vaccinations were indicated to also be beneficial to oncological population. However, data about the impact of anticancer-treatments and the timing between vaccinations and systemic therapy delivery were not available. We aimed to evaluate potential factors influencing the outcome of the COVID-19 vaccination in cancer patients. We prospectively collected data of patients undergoing the COVID-19 vaccination with gastro-entero-pancreatic and neuroendocrine neoplasms, treated at our institute, between 03/2021 and 12/2021. We enrolled 46 patients, 63.1% males; at the time of data collection, 86.9% had received two-doses of Pfizer-BioNTech and the rest had received the Moderna vaccine. All patients obtained a subsequent immune-response. Chemotherapy seems to determinate a significantly lower antibody response after vaccination compared to the other anti-cancer agents (*p* = 0.004). No significant effect on immune-response was reported for both vaccinations performed ≤7 vs. >7 days from the last systemic treatment (*p* = 0.77) and lymphocytes count (*p* = 0.11). The findings suggest that the optimal timing for COVID-19 vaccination and lymphocytes count are not the issue, but rather that the quality of the subset of lymphocytes before the vaccination determine the efficacy level of immune-response in this population.

## 1. Introduction

Since December 2019, reported in Wuhan (China) and then all over the world, Severe Acute Respiratory Syndrome Coronavirus-2 (SARS-CoV-2) dramatically distressed the balance of different health authorities, leading the World Health Organization (WHO) to declare it a pandemic in March 2020 [1]. Since then, over the last 2 years, coronavirus disease-19 (COVID-19) caused more than 500 million infections and over 6 million deaths worldwide [2].

The emergency caused by the pandemic led to crucial changes in healthcare management that dramatically impacted patients’ care: elective surgery, screening/follow-up diagnostic assessments [3], and access to non-urgent treatments were restricted or withheld to allocate resources and personnel to COVID-19 outbreak management [4,5,6].

Specifically, for oncologists and cancer patients, the COVID-19 outbreak led to delays in diagnosis and treatments [7], with a negative prognostic impact due to the detection of cases at a more advanced stage [8]. 

Moreover, despite the efforts made to maintain COVID-free oncological departments, the indications on the logistic management, and the planning of therapies for cancer patients [9], the pandemic heavily affected oncological patients. A higher risk of death and severe complications have indeed been evidenced in cancer patients compared to the general population, and treatment delay/interruption frequently occurred [10,11]. 

The introduction of COVID-19 vaccination at the end of 2020 deeply changed the clinical scenario of SARS-CoV-2 infection, with cutback of infections, reduction in viral load, and severe complications [12]. Owing to international efforts, several vaccines were developed, including mRNA-based, peptide, and inactivated vaccines. In Italy, two mRNA-based vaccines (Pfizer mRNA BNT162b2 and COVID-19 Vaccine Moderna mRNA-1273) have been approved, two non-replicating viral vector vaccines (Vaxzevria—ex COVID-19 AstraZeneca vaccine and Jcovden—ex COVID-19 Janssen vaccine), and one peptide vaccine (COVID-19 Nuvaxovid—Novavax) [13]. International guidelines immediately agreed on the benefit of SARS-CoV-2 vaccination for high-risk subjects, including the whole oncological population. However, despite the unconfutable benefit of vaccinations, indications relied on previous statements about other vaccinations in cancer patients [9,14]. In detail, evidence regarding the impact of anticancer treatment administration and the timing between vaccinations and treatment delivery were very limited, leaving physicians to choose according to the individual therapeutic scenarios.

In this evolving situation, we designed the VACCINATE trial, an observational study aimed to evaluate the timing of the SARS-CoV-2 vaccination in cancer patients receiving systemic treatments and potential factors influencing the efficacy and safety of COVID-19 vaccines. 

## 2. Materials and Methods

### 2.1. Patients and Methods

The VACCINATE is a single-center, observational, prospective study performed between March 2021 and December 2021. We prospectively collected data of consecutive oncological patients with solid tumors, managed, and treated at the Gastrointestinal and Neuroendocrine Tumors Division of the European Institute of Oncology (IEO), Milan. We considered eligible for the study all patients ≥ 18 years (y) diagnosed with a gastrointestinal, (GI) hepato-biliary-pancreatic (HBP), or neuroendocrine neoplasm (NEN), receiving an ongoing anticancer treatment and a concomitant SARS-CoV-2 vaccine (first or second dose). All types of the vaccine were approved for cancer patients according to clinical practice; Moderna/SpikeVax [15] and Pfizer-BioNTech [16] were eligible for the purpose of the study.

For each patient, we collected demographic and clinical data, as well as the type and timing of the SARS-CoV-2 vaccination in addition to collecting tumor information including histology, pathological features, stage, grading, setting of treatment (adjuvant, neoadjuvant, first line for advanced disease, subsequent lines) and timing of therapy administration compared to timing of the vaccination. All data were collected in an electronic medical case report form (eCRF) and stored in a web-secured database (REDcap system). All patients underwent blood sampling to determine their lymphocyte count within 14 days before each dose of the vaccine; another blood sample for the assessment of SARS-CoV-2 IgG antibody response was collected after 15 ± 5 days from the vaccination, and then every 40 ± 10 days from the previous blood draw. For patients with a previous documented SARS-CoV-2 infection, a further IgG dosage was performed before vaccination. Blood samples for the immune response assessment were centrally analyzed at the laboratory of the Monzino Cardiologic Center, Milan, while complete blood cell counts were performed at the IEO laboratory. The SARS-CoV-2 antibodies titers were assessed by means of the DiaSorin LIAISON^®^ SARS-CoV-2 trimeric test that detected anti-trimeric S spike protein IgG antibodies with sensitivity and specificity of 98.7% and 99.5%, respectively [17]. The IgG titers were expressed in AU/mL and the diagnostic cut-off of the test was 13 AU/mL. Additionally, patients received a questionnaire for reporting the most frequent adverse events. The patients indicated any health-related changes within 2 weeks of the vaccination, their duration, and their consequences (e.g., sequelae/resolution). Toxicity data from anticancer treatments after the vaccine dose were prospectively collected.

All patients provided written, informed consent. The study was approved by the IEO and Monzino’s internal medical ethics committee (IUD: IEO 1525) and performed in accordance with the principles of the Declaration of Helsinki, Good Clinical Practice guidelines, and applicable government regulations.

The primary endpoint of the study was to collect data on the antibody response to SARS-CoV-2 vaccination in cancer patients. Secondly, we aimed to correlate the antibody response to the clinical, pathological, and biological features, in order to identify data relevant for the management of oncologic patients during the pandemic.

### 2.2. Statistical Methods

We calculated the median and interquartile range (IQR) for continuous variables and absolute and relative frequencies as summary measures of categorical variables. Based on the nature of variables, Fishers–Exact tests, Wilcoxon rank-sum tests, or the Kruskal–Wallis rank sum tests were performed. A multivariable logistic model was applied to identify independent factors associated with AEFI (Adverse Events Following Immunization). A multivariable mixed-effects model was adopted to analyze changes in IgG and associated factors over time. Normal distribution of residuals from fully adjusted models were graphically checked and log transformation was adopted when it was needed to achieve normality. All reported values were two sided and *p* < 0.05 was considered statistically significant. All analyses were carried out using the R studio (R version 4.0.2).

## 3. Results

**Figure 1:**
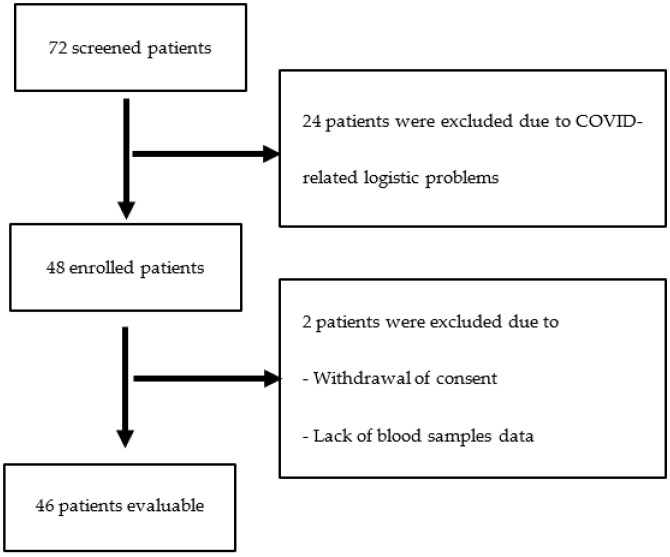
Flowchart of enrollment of patients.

In total, we enrolled 48 patients, but only 46 were considered in the final analysis due to consent withdrawal in one case and lack of immune-response data in another (Figure 1). 

From 72 patients with gastro-entero-pancreatic and neuroendocrine neoplasms who were treated at our institute between March and December 2021 and underwent the COVID-19 vaccination, 24 were excluded due to COVID-19 logistic problems (e.g., the patients were unable to leave the hospital for the exams planned in the trial). Another 2 patients were excluded due to a withdrawal of consent and lack of data about the immune response. Therefore, forty-six patients were evaluated for the final analysis.

Altogether, 29 patients were male (63.1%) and the median age was 57 years (y) (range 47–64.75). The most represented tumor was colorectal cancer (28.3%) followed by GEP-NET (26.0%) and gastric cancer (15.2%). Table 1 reports the features of the population. 

Around three-quarters of the population had a metastatic disease (*n* = 34, 73.9%) and 42 (91.3%) patients were receiving an active antitumoral therapy over the study. The overwhelming majority of patients (*n* = 40, 86.9%) received two-doses of the Pfizer-BioNTech vaccine, whereas five patients received the Moderna vaccine. Four patients (8.7%) did report a previous SARS-CoV-2 infection, and 37 (75%) had relevant comorbidities other than cancer (Table 1). After both vaccination doses, 12/46 (26%) reported any grade adverse event (all <Grade 3 according to the Common Terminology Criteria for Adverse Events v 5.0) and 7/46 (15.2%) reported any grade adverse after the second dose (all <Grade 3). Local effects including pain, oedema, and rash on injection site were the most common adverse event, followed by fever and asthenia.

The median baseline lymphocytes value was 1530 (range 1180–1940) and 1405 (1267–1870) one week after the vaccination. The median antibodies after 1 dose were 38.65, while after 2 vaccine doses, the median antibodies were 7344.8 AU/mL (3450.8–16,759). 

**Figure 2:**
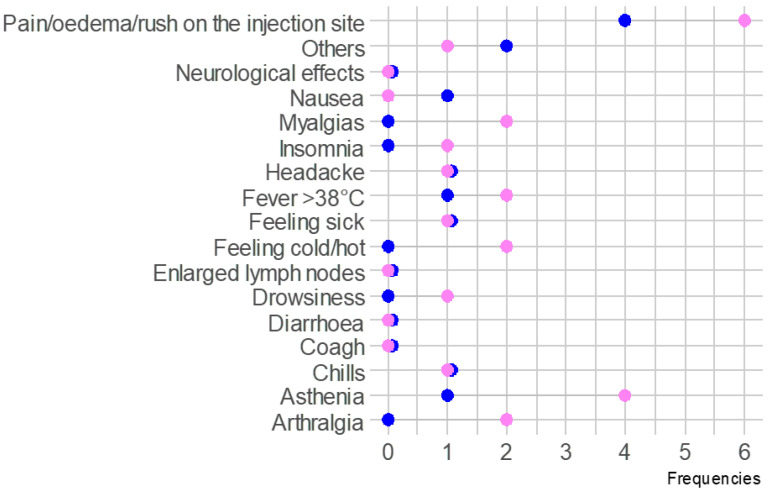
Lollipop chart of adverse events after SARS-CoV-2 vaccination between males (in blue) and females (in pink).

The multivariate analysis, adjusted for age and lymphocytes values, showed a trend to a higher risk of adverse events in female patients compared to males (*p* = 0.059) (Figure 2). 

No significant differences were reported between males and females in terms of adverse events.

**Figure 3:**
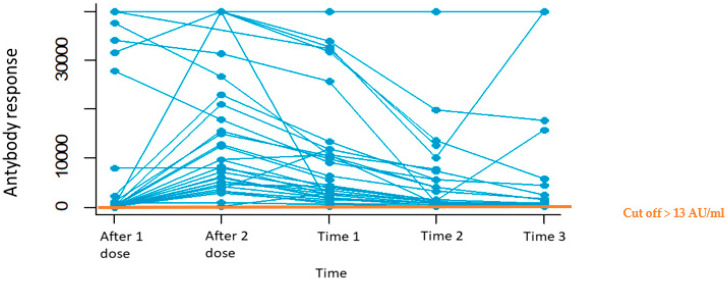
Trend of the antibody response after 1 and 2 doses of the SARS-CoV-2 vaccine and at the following default times (40 ± 10 days from the last blood test).

All patients obtained an immune response to the SARS-CoV-2 vaccine (Figure 3), represented by an IgG titer greater than 13 AU/mL. 

All the patients reported an immune response after the COVID-19 vaccination.

**Figure 4:**
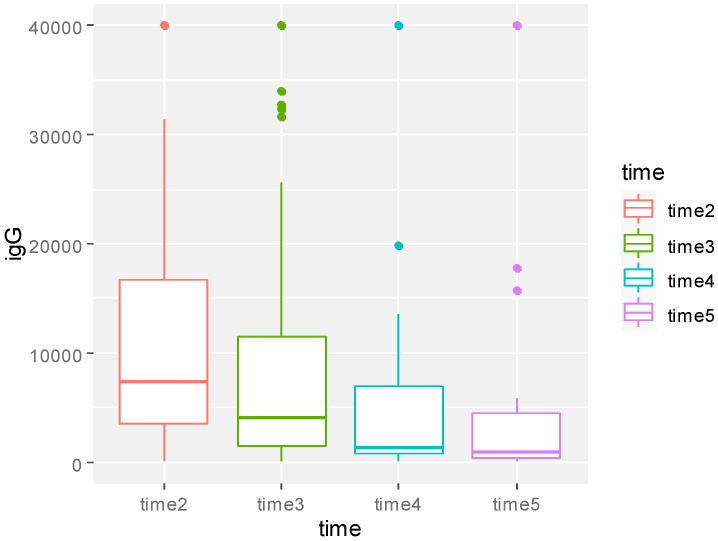
Boxplot of anti-SARS-CoV-2 IgG values over time of the whole sample at the different times of observation. Times 1, 2, and 3 refer to the moment of further blood assessment (40 ± 10 days from the previous). *p*-value obtained from multivariable model for repeated measure analysis adjusted for age, therapy, time, and IgG after first dose.

We observed significantly lower values of IgG for patients treated with chemotherapy compared with patients receiving other anti-cancer agents (*p* = 0.004) (Figure 4). 

However, no significant effect on immune response was reported for vaccinations performed within 7 days vs. over 7 days from the last systemic treatment (*p* = 0.77). At the time of study entry, none of the patients developed a SARS-CoV-2 infection after the vaccination.

**Figure 5:**
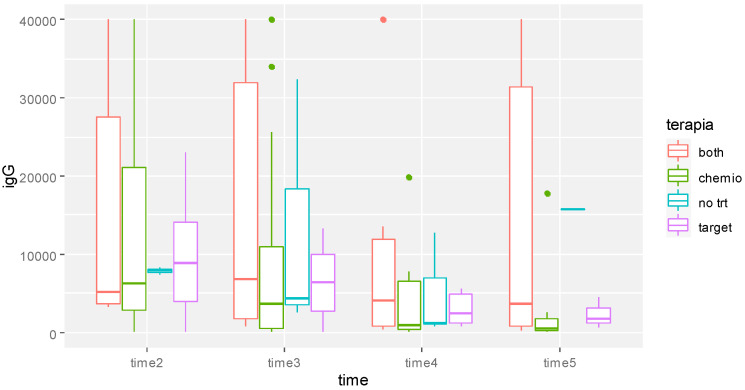
Boxplot of anti-SARS-CoV-2 IgG values of whole patients’ samples over time by therapy. Times on the Y-axis Y refer to 40 ± 10 days blood assessment after the previous. *p*-value obtained from multivariable model for repeated measure analysis adjusted for age, therapy, time, and IgG (AU/mL) after first dose.

**Figure 6:**
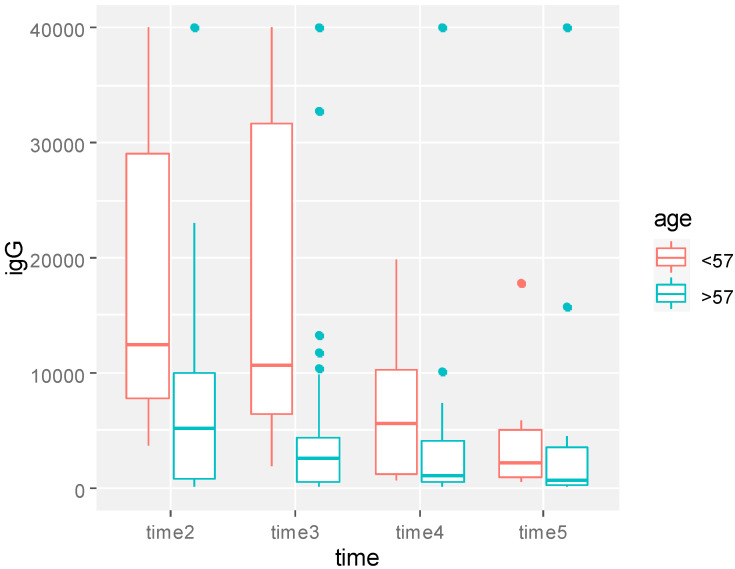
Boxplot of SARS-CoV-2 IgG values over time by age at diagnosis. *p*-value obtained from multivariable model for repeated measure analysis adjusted for age, therapy, time, and IgG after first dose.

The analysis reported a significant decrease of the immune response during the observation (*p* = 0.01) (Figure 5), with higher anti-SARS-CoV-2 IgG values for patients ≤ 57 vs. >57 y of age (*p* = 0.002) (Figure 6).

## 4. Discussion

Compared to the first dramatic phase of the COVID-19 pandemic, when only symptomatic and supportive measures could be applied to reduce mortality, the introduction of efficient SARS-CoV-2 vaccines radically improved the outcomes of patients, including patients with cancer.

In this context of fast-emerging indications, our prospective study confirmed the efficacy and safety of vaccinations in cancer patients receiving systemic treatments, even though chemotherapy was significantly associated with lower levels of SARS-CoV-2 antibodies. However, we interestingly observed that the induction of an immune response was not affected by a close timing of systemic treatment administration and vaccination. Despite the low number of subjects, our study could provide meaningful clinical insights for oncologic patients management [18,19]. 

Several studies reported the efficacy and safety of COVID-19 vaccinations for oncologic patients. The VOICE trial was a multicenter study [20] that prospectively analyzed the efficacy and safety of the Moderna Spikevax vaccine in patients with solid tumors who were treated with chemotherapy, immunotherapy, or combinations. Almost the whole evaluable population showed an immune response to the vaccination (736/743, 99%) with a manageable toxicity profile. According to the Center of Disease Control and Prevention (data updated on 21 June 2022—https://www.cdc.gov/coronavirus/2019-ncov/vaccines/), local reactions at the site of the injection are expected in almost 85% of adult subjects after the first dose of the COVID-19 vaccination, with a trend to a lower percentage after the second dose. The systemic events are instead expected in almost 77% of subjects after the COVID-19 vaccination, with a trend to lower manifestations after the first rather than the second dose. In both the situations, severe adverse events are expected in almost 0.6% of the vaccinated population, maintaining a good and well-manageable toxicity profile [13]. 

Even if we only used a small sample size, our real-life analysis prospectively showed a lower incidence of adverse events after the first and the second dose of vaccinations compared to the whole population and, similarly to other studies, local adverse events at the injection site were the most common [21]. One explanation of this phenomenon might be that oncological patients tend not to report effects that are negligible, compared to cancer and treatment-related consequences, unless they interfere with their quality of life. Another reason why our population showed less adverse events compared to the whole population may be related to a lower reactogenicity due to the immunosuppressive systemic treatments that they were receiving. Regarding the latter possibility, instead, our study prospectively confirmed the efficacy of Moderna and Pfizer-BioNTech vaccines in this specific gastrointestinal and neuroendocrine neoplasms population. Despite a moderately short follow-up, none of our patients developed a COVID-19 infection after vaccination at the time of the data analysis. Other studies reported comparable results, showing a significant antibody titer after the vaccine second dose in 83–86% of cancer patients [22,23]. 

Despite the encouraging data, the lack of indications on the optimal timing of the SARS-CoV-2 vaccination forced the oncological community to decide when to vaccinate the patients based on the individual patient’s therapeutic scenario, rather than with a shared and standardized approach. Indeed, the international guidelines recommended vaccinating patients with solid or hematologic malignancies at the earliest available opportunity [24]. For most solid tumors, the indicated timing should be drug-mechanism based; according to studies on flu, the optimal timing for patients treated with chemotherapy should be to administer the SARS-CoV-2 vaccine 1–2 weeks before or after the systemic therapy, to allow an adequate immune response. According to the results of our analysis, the age of patients and the type of systemic treatment seemed to be the only factors influencing the anti-COVID-19 IgG levels. A possible reason why aging may be associated with lower immune response after vaccination is that immunosenescence and inflamm-aging, given by a progressive change in proinflammatory factors and a remodulation of the immune system pattern, play the main role in reducing the immune response and worsening the viral infections’ outcome [25]. For chemotherapy, as reported in previous studies, a lymphocytes count reduction seemed to be the most impacting factor on immune response [26,27,28]. Anyway, most of these studies concerned hematologic malignancies and reported a higher vulnerability for those patients receiving CD20 B-cell depletion treatments. On the other hand, different methods of analysis and the lack of reference standards render the results of the few studies on solid tumors debatable and uncertain to apply in clinical practice.

The recent paper by Nelli F et al. analyzed the subset of peripheral lymphocyte counts and the SARS-CoV-2 immune response after vaccination and reported a significant impact of chemotherapy and corticosteroids on T helper and B lymphocyte counts [29]. 

However, although chemotherapy was associated with a lower immune-response to SARS-CoV-2 vaccination compared to the other treatments in our analysis, no significant correlation has been reported either between peripheral lymphocytes count and anti-COVID-19 IgG levels or between the timing of the therapies administration and antibody titers. A possible explanation is that the small sample size is a limitation of this study. Another explanation may be that not all of the regimens used to treat our population are associated with a real lymphocytes dysfunction. In fact, the sample consisted of NEN patients treated with temozolomide and others previously receiving peptide receptor radioligand therapy (PRRT), which often results in deep, long-lasting low lymphocyte levels [30,31,32,33,34]. 

On the other hand, our sample also included GI patients receiving platinum-based chemotherapy, which instead seems to induce active phenotypes and functions of CD8 tumor-infiltrating T lymphocytes, supporting a chemo-immunotherapy combination [35]. Therefore, according to these observations and our study results, a lower immune response in solid cancer patients might be associated with the impact of different therapeutic regimens on the phenotype of the lymphocytes subset, irrespective of the overall lymphocytes count. These findings are comparable to data on the timing of flu vaccinations during chemotherapeutic treatments which showed no significant differences on antibody responses between patients concurrently inoculated with chemotherapy and those inoculated during the lymphopenia period [36]. 

Our study presents several limitations. First of all, due to the urgency of the pandemic and the vaccination program, the sample of our analysis was small and heterogeneous in terms of treatments and disease biology, involving patients with only gastrointestinal carcinomas or neuroendocrine neoplasms. Second, we did not have the possibility to analyze the different lymphocytic subpopulations that may have allowed a better interpretation of our findings. Lastly, the short follow-up did not permit an evaluation of the protection induced by the vaccination against the new different SARS-CoV-2 viral strains. At the same time, the heterogeneity of the sample may be particularly representative of the real-life experience and provide insights and hypotheses for further analyses. 

## 5. Conclusions

To our knowledge, this is the first prospective analysis focused on the SARS-CoV-2 vaccination timing in patients with solid cancers on systemic antitumor treatments. No significant impact on the count or timing of the systemic treatments administration of anti-COVID-19 IgG levels of the peripheral lymphocytes has emerged, while chemotherapy and age > 57 y seems to influence the immune response to the SARS-CoV-2 vaccination. These results suggest that the timing of the COVID-19 vaccination and lymphocytes count are not the issue, but rather that the functionality of the lymphocytic subsets before the vaccination can determine the efficacy level of the vaccines in solid cancers patients. Further prospective studies are needed to address this topic.

## Figures and Tables

**Table 1:** Main subject and tumor characteristics (*n* = 46).

Age at Diagnosis, Median (IQR)		57 (47–64.75)
Sex, *n* (%)	Female	17 (36.9)
	Male	29 (63.1)
Comorbidities, *n* (%)	No	9 (25)
	Yes	37 (75)
	Type of comorbidities:	
	PTE/DVT	7 (18.9)
	Other cancers	8 (21.6)
	Metabolic	12 (32.4)
	Cardiovascular	13 (35.1)
	Autoimmune disease	1 (2.7)
	Others	28 (75.7)
Previous COVID-19, *n* (%)	No	42 (91.3)
	Yes	4 (8.7)
	Esophagus	2 (4.3)
	Stomach	7 (15.2)
	Colorectal	13 (28.3)
Tumor, *n* (%)	Anus	1 (2.2)
	Pancreas (adenocarcinomas)	3 (6.5)
	Biliary tract	1 (2.2)
	NEN	19 (41.3)
	GEP-NET	12 (63.1)
	GEP-NEC	2 (10.5)
	Thoracic-NET	2 (10.5)
	Thoracic-NEC	1 (5.3)
	Other NEC	2 (10.5)
	Localized/locally advanced	7 (15.2)
Current stage, *n* (%)	Distant metastatic	34 (73.9)
	No evidence of disease	5 (10.9)
Active antitumor therapy, *n* (%)	No	4 (8.7)
	Yes	42 (91.3)
	SSA (somatostatin analogue)	3 (3.8)
	anti-mTOR	2 (2.6)
	Chemotherapy Platin	15 (19.2)
	Etoposide	2 (2.6)
	Fluoropyrimidines	28 (35.9)
	Irinotecan	8 (10.2)
Type of active antitumor therapy, *n* (%)	Temozolomide	2 (2.6)
	Immune checkpoint inhibitors	4 (5.1)
	Anti-VEGFR	7 (9.0)
	Gemcitabine	1 (1.3)
	Taxane	5 (6.4)
	Other	1 (1.3)
	Hormonal therapy	7 (15.2)
	Chemotherapy	21 (45.6)
Previous types of Systemic treatments, *n* (%)	Targeted therapy	12 (26.1)
	PRRT	7 (15.2)
	Pfizer	40 (86.9)
Vaccine type dose 1, *n* (%)	Moderna	5 (10.9)
	Missing	1 (2.2)
Vaccine type dose 2, *n* (%)	Pfizer	37 (80.4)
	Moderna	3 (6.6)
	Missing	6 (13.0)
Lymphocytes at baseline, median (IQR)		1530 (1180–1940)
Antibody response after 1 dose, median (IQR)		38.65 (21–3679.6)
Lymphocytes after 1 dose, after 2 dose median (IQR)		1405 (1267–1870)
Antibody response after 2 dose, median (IQR)		7344.8 (3450.8–16,759)

PTE/DVT: pulmonary thromboembolism/deep veins thrombosis; NEN: neuroendocrine neoplasm; GEP: gastro-entero-pancreatic; NET: neuroendocrine tumor; NEC: neuroendocrine carcinoma; SSA: somatostatin analogue; PRRT: Peptide Radionuclide Receptor Therapy.

## Data Availability

IEO shall be classified as autonomous Data Controllers pursuant to Regulation (EU) 2016/679 of the European Parliament and Council of 27 April 2016 (GDPR).
